# Correlating preoperative imaging with intraoperative fluoroscopy in iliosacral screw placement

**DOI:** 10.1007/s10195-015-0363-x

**Published:** 2015-07-21

**Authors:** Jonathan G. Eastman, Milton L. Chip Routt

**Affiliations:** Department of Orthopaedic Surgery, University of California, Davis Medical Center, 4860 Y Street, Suite 3800, Sacramento, CA 95817 USA; Department of Orthopaedic Surgery, University of Texas, Health Sciences Center at Houston, Houston, TX USA

**Keywords:** Iliosacral screw, Preoperative planning

## Abstract

**Background:**

Percutaneous iliosacral screw placement can successfully stabilize unstable posterior pelvic ring injuries. Intraoperative fluoroscopic imaging is a vital component needed in safely placing iliosacral screws. Obtaining and appropriately interpreting fluoroscopic views can be challenging in certain clinical scenarios. We report on a series of patients to demonstrate how preoperative computed tomography (CT) imaging can be used to anticipate the appropriate intraoperative inlet and outlet fluoroscopic views.

**Materials and methods:**

24 patients were retrospectively identified with unstable pelvic ring injuries requiring operative fixation using percutaneous iliosacral screws. Utilizing the sagittal reconstructions of the preoperative CT scans, anticipated inlet and outlet angle measurements were calculated. The operative reports were reviewed to determine the angles used intraoperatively. Postoperative CT scans were reviewed for repeat measurements and to determine the location and safety of each screw.

**Results:**

Preoperative CT scans showed an average inlet of 20.5° (7°–37°) and an average outlet of 42.8° (30°–59°). Intraoperative views showed an average inlet of 24.9° (12°–38°) and an average outlet of 42.4° (29°–52°). Postoperative CT scans showed an average inlet of 19.4° (8°–31°) and an average outlet of 43.2° (31°–56°). The average difference from preoperative to intraoperative was 4.4° (−21° to 5°) for the inlet and 0.45° (−9° to 7°) for the outlet. The average difference between the preoperative and postoperative CT was 2.04° (0°–6°) for the inlet and 2.54° (0°–7°) for the outlet.

**Conclusion:**

There is significant anatomic variation of the posterior pelvic ring. The preoperative CT sagittal reconstruction images allow for appropriate preoperative planning for anticipated intraoperative fluoroscopic inlet and outlet views within 5°. Having knowledge of the desired intraoperative views preoperatively prepares the surgeon, aids in efficiently obtaining correct intraoperative views, and ultimately assists in safe iliosacral screw placement.

**Level of evidence:**

IV, Retrospective case series.

## Introduction

Percutaneous iliosacral screw fixation of unstable posterior pelvic ring injuries has become a common successful treatment method [1–4]. In order to place iliosacral screws safely, a thorough understanding of the possible osseous fixation pathways is paramount [5, 6]. Recognizing sacral dysmorphism and accommodating anatomic variations of the posterior pelvic ring requires detailed knowledge of the osteology [7–11]. In addition to obtaining an accurate reduction, combining the osteological details with the corresponding intraoperative fluoroscopic imaging is necessary to safely perform percutaneous fixation. Inlet and outlet fluoroscopic views are utilized to safely place iliosacral screws. An intraoperative lateral fluoroscopic view can be extremely helpful by providing a third dimension that helps verify the osteology seen on the inlet and outlet views [1–3, 12, 13].

**Fig. 1:**
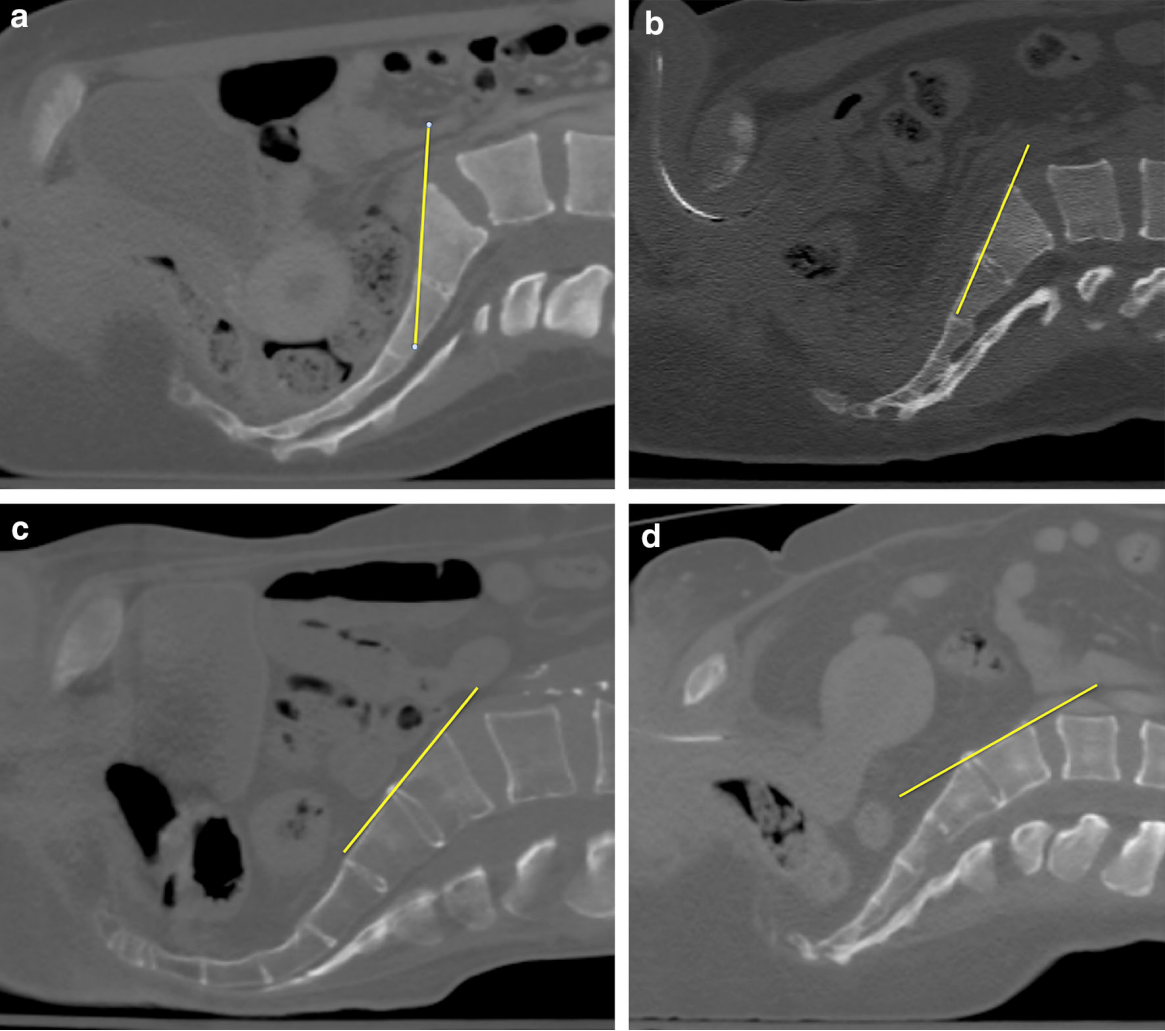
Preoperative CT scan with sagittal reconstruction. The image has been rotated 90° from vertical to simulate the patient lying supine on the operating room table. The *yellow line* parallels the anterior cortex of the S1 body with which the fluoroscopic beam would parallel for an inlet view. The anatomic variability of the posterior pelvic ring is demonstrated above in four different patients. Note the near vertical orientation of the S1 sacral body in **a**. There is a gradual increase in the lordotic alignment in **b** and the S1 body is nearly horizontal in **d** (color figure online)

The varying degrees of sacral kyphosis or lordosis as well as the presence of any degree of sacral dysmorphism leads to a wide range of angles required to achieve appropriate inlet and outlet radiographs as well as intraoperative fluoroscopic views [14–16] (Fig. 1). In addition to the details of the fracture, the preoperative CT scan can be used to measure the ideal inlet and outlet angles. These measurements can be taken to the operating theater to help obtain the appropriate fluoroscopic views. This process can help surgeons quickly obtain satisfactory intraoperative imaging and in attaining adequate imaging for all patients. This could be very helpful in difficult clinical situations including morbid obesity, bowel gas, and the presence of contrast. The purpose of this study was to determine whether the anticipated inlet and outlet angles obtained from preoperative CT scans are the same angles utilized with intraoperative fluoroscopy. We hypothesize that preoperative CT imaging can successfully be used to accurately plan and anticipate the exact inlet and outlet angles actually used intraoperatively during percutaneous iliosacral screw fixation of unstable posterior pelvic ring injuries.

## Materials and methods

After obtaining Institutional Review Board approval, a 3-month review was performed from our prospectively collected trauma database at a regional level 1 trauma center. This database has recorded all operatively managed fractures since 1989. Fractures are entered and coded according to the Arbeitsgemeinschaft für Osteosynthesefragen/Orthopaedic Trauma Association (AO/OTA) Fracture Classification System by orthopaedic trauma fellows trained in this classification system [17]. Data is stored and manipulated using a commercially available software program (Microsoft Access). Inclusion criteria required skeletal maturity, a complete medical chart relative to their injury, adequate preoperative and postoperative radiographic imaging including CT scans, and to have had their definitive surgical procedure performed at our center. From 29 May 2012 to 31 July 2012, 24 consecutive patients with unstable pelvic ring injuries who underwent operative fixation using percutaneous iliosacral screws were identified. Pelvic ring injuries and associated instability was identified by preoperative radiographic and CT imaging which demonstrated combinations of anterior and posterior pelvic ring disruptions. Anterior ring injuries consisted of either unilateral or bilateral superior and inferior rami fractures or complete symphysis pubis disruptions. Posterior pelvic ring injuries consisted of a complete sacral fracture, sacroiliac joint disruption, or a posterior ilium/sacroiliac joint fracture dislocation. All patients underwent an examination under anesthesia as previously described [18]. These examinations demonstrated and documented the instability present, especially in the 61-B injury patterns. All patients had documented posterior pelvic ring instability and therefore underwent appropriate operative fixation with accompanying percutaneous fixation to stabilize the posterior aspect of their pelvic ring injury. One orthopaedic traumatologist at a regional level 1 trauma center treated all patients. All iliosacral screws were placed using standard and previously described techniques with inlet, outlet, and lateral fluoroscopic imaging only utilizing a C-arm [2, 19, 20]. Once adequately resuscitated and evaluated, each patient was sedated and transported to the operating suite where they surrendered to general anesthesia. The patient was then transferred onto a radiolucent operating table and placed in a supine position. The patient was placed onto two folded blankets beneath the lumbosacral spine. The blanket bump is precisely placed with the distal aspect of the blankets at the testicles or labia and in the center of the lumbosacral spine. The perineum was cleansed and isolated from the operative field with adhesive drapes. The entire abdomen and bilateral flanks were then sequentially cleansed with iodine and isopropyl alcohol. Accurate reductions of the pelvic ring injuries were achieved by both open and closed means as guided by the injury patterns and surrounding soft tissue status. All screws placed in this cohort were with the patient in a supine position. Posterior ring fixation consisted of 7.0-mm diameter cannulated screws (Synthes, Paoli, PA, USA) or 7.0-mm diameter cannulated screws (Zimmer, Warsaw, IN, USA) of varying length; both fully and partially threaded screws were used depending on injury pattern, available osseous fixation pathways, and associated fixation strategy. Each patient’s chart was reviewed for patient gender, age, mechanism of injury, and AO/OTA injury classification.

**Fig. 2:**
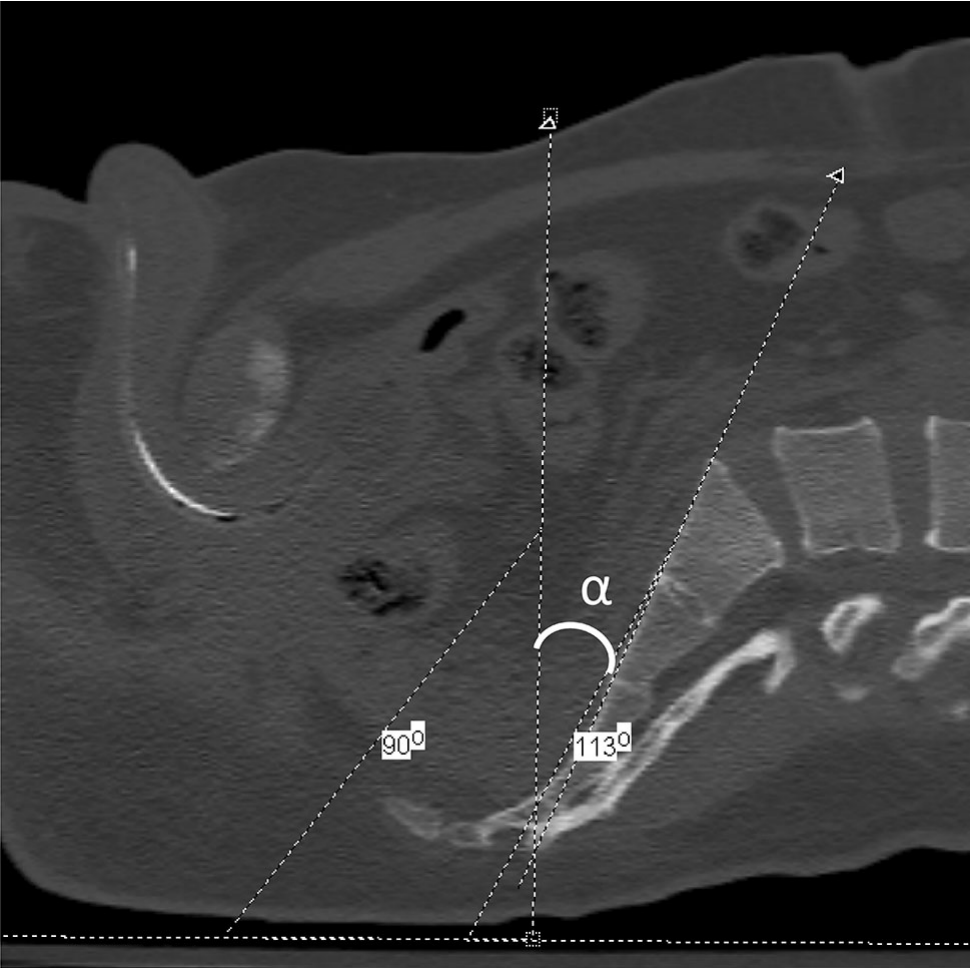
Preoperative CT scan with sagittal reconstruction at the midline demonstrating the anticipated inlet measurement. The *horizontal line* parallels the surface the patient is lying on. The *oblique line* parallels the anterior surface of the S1 body. The line at 90° simulates a straight up and down position of the C-arm. The anticipated inlet angle, labeled *α*, would therefore be 23° (113°−90°) of cephalad tilt of the C-arm

**Fig. 3:**
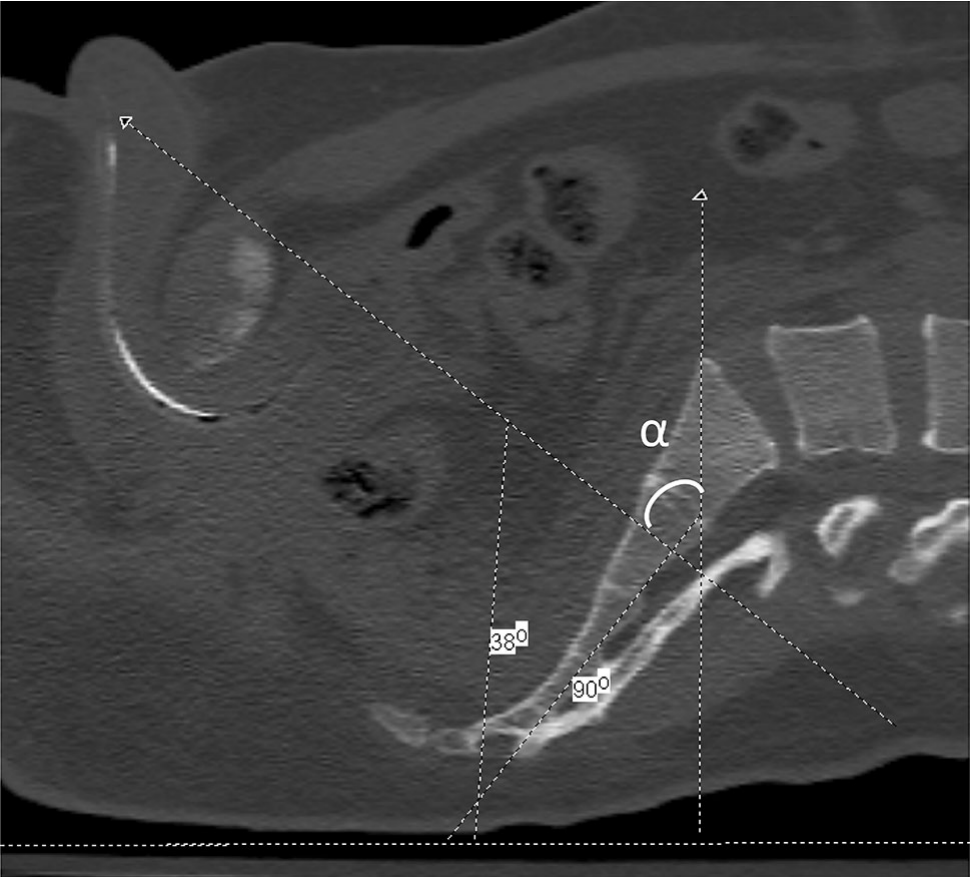
Preoperative CT scan with sagittal reconstruction at the midline demonstrating the anticipated outlet measurement. The *horizontal line* parallels the surface the patient is lying on. The *oblique line* overlaps the superior aspect of the symphysis pubis to the S2 body. The line at 90° simulates a straight up and down position of the C-arm. The anticipated outlet angle, labeled *α*, would therefore be 52° (90°−38°) of caudal tilt of the C-arm

The preoperative CT scan of each patient was reviewed using a picture archiving and communication system (PACS) using Centricity Version 2.1 (GE Medical Systems, Waukesha, WI, USA). Utilizing the midline view of the sagittal reconstruction images, inlet and outlet angle were calculated. One surgeon made both the preoperative and postoperative CT measurements. The process to make the measurement included rotating the entire image 90° clockwise to simulate a supine position on the operating table. A horizontal line parallels that surface of the CT gantry. This line simulates how the patient will be lying supine on the operating table. A line is placed at 90° to the horizontal line that simulates a straight up and down position of the C-arm that would produce an anteroposterior (AP) view. The inlet view angle is measured as a line that parallels the anterior cortex of the S1 body in reference to the horizontal line. The anticipated inlet angle would be the difference in angles from the straight up and down position of the C-arm down to the angle measured to obtain an image that parallels the anterior cortex of the S1 body (Fig. 2). For the outlet view, the same horizontal and 90° lines are drawn as noted above. The outlet angle for an S1 iliosacral screw is drawn as the line that overlaps the symphysis over the center of the S2 body. The anticipated outlet angle would then be the difference between the straight up and down position down to the angle measured to obtain an image placing the superior symphysis overlying the center of the S2 body (Fig. 3) [16, 19].

The operative reports of each patient were subsequently reviewed to determine the inlet and outlet angles used intraoperatively. The difference between the preoperative anticipated inlet and outlet angles and intraoperative fluoroscopic inlet and outlet angles was determined. The fluoroscopic angle was defined as the center value and the preoperative value was either less than (negative number) or greater than (positive number) that value. A postoperative CT was obtained on each patient within 24 h from surgery. This is standard treatment protocol and verifies reduction and implant placement. Each postoperative CT was reviewed to repeat the inlet and outlet angle measurements without direct knowledge of the previously measured angles. The preoperative and postoperative measurements were made by a single surgeon to assess whether the method of measurement was reproducible. The location and safety of each iliosacral screw was determined and each screw was defined as intraosseous, juxtaforminal, or extraosseous. An intraosseous position was defined by the presence of cancellous bone completely surrounding the screw on all CT cuts. Juxtaforaminal was defined by a lack of cancellous bone surrounding the screw but an intact cortical rim at the ala, S1 or S2 neuroforaminal tunnel, and the spinal canal. Extraosseous was defined as any evidence of cortical discontinuity. Postoperative rehabilitation and mobilization were guided by each patient’s musculoskeletal injuries and overall medical condition under the direct supervision of licensed physical therapists using standard protocols. Statistical analysis was performed using paired *T*-tests for comparison of the preoperative CT measurements to the intraoperative fluoroscopic measurements as well as the Pearson product-moment correlation coefficient for assessment of intraobserver reliability in obtaining the preoperative and postoperative CT measurements.

## Results

The cohort consisted of 24 patients (14 males and 10 females) with an average age of 47.7 years (20–82). The mechanisms of injury included eight patients with falls, five patients involved in motor vehicle collisions, four patients sustaining equestrian injuries, three patients involved in motorcycle collisions, three patients involved in automobile versus pedestrian accidents, and one patient who sustained a crush injury. AO/OTA classification showed five 61-B injury patterns—two 61-B1.1, two B2.1, and one B3.2. There were 19 61-C injury patterns—three 61-C1.2, seven 61-C1.3, two 61-C2.3, one 61-C3.1, two 61-C3.2, and four 61-C3.3. Two patients also sustained accompanying acetabular fractures. One patient sustained an open pelvic ring injury with complete symphyseal disruption and complete sacral fracture medial to the neuroforaminal tunnels. The open wound included his scrotum and perineum and was managed with multiple irrigation and debridements, closure of his scrotal wound, and packing to closure of his perineal wound. His posterior pelvic ring underwent closed reduction and percutaneous fixation and his anterior ring injury was treated with external fixation for 6 weeks. The average time until surgery was 4.4 days (1–28). Twenty-two patients were managed with closed reduction. Two patients required an open reduction of their displaced sacroiliac joint dislocations through an anterior approach. Of 24 patients, 9 (37.5 %) had some degree of sacral dysmorphism as previously defined [7, 11].

Utilizing the measurement method described above, preoperative CT scans showed an average inlet view of 20.5° (7°–37°) and an average outlet view of 42.8° (30°–59°). The intraoperative fluoroscopic views showed an average inlet of 24.9° (12°–38°) and an average outlet view of 42.4° (29°–52°). Postoperative CT scans showed an average inlet of 19.4° (8°–31°) and an average outlet of 43.2° (31°–56°), (Table 1). The average difference between the preoperative to intraoperative inlet view was 4.4° (−21° to 5°), which was statistically significant with *p* = 0.0003. The average difference between the preoperative to intraoperative outlet view was 0.45° (−9° to 7°), which was not statistically different. The average difference when comparing the preoperative and postoperative CT scans was 2.04° (0°–6°) for the inlet and 2.54° (0°–7°) for the outlet view (Table 2). In comparing the preoperative and postoperative CT inlet and outlet angle measurements, the Pearson product-moment correlation coefficient reached a correlation of >0.9. The inlet and outlet measurement angles for each patient for the preoperative CT scan, fluoroscopic views, and postoperative CT scan are listed in Table 3. A total of 58 iliosacral screws were placed in this patient cohort. Postoperative CT imaging demonstrated that 51 of 58 screws (88 %) were intraosseous and 7 of 58 screws (12 %) were juxtaforaminal. There were no extraosseous screws. Of the 58 screws, 31 (53 %) were iliosacral-style screws and 27 (47 %) were considered transiliac transsacral screws. Of the 31 iliosacral screws, 4 (12.9 %) were noted to be juxtaforaminal and of the 27 transiliac transsacral screws, 3 (11 %) were noted to be juxtaforaminal.

**Table 1:** Inlet and outlet angles obtained using preoperative and postoperative sagittal CT imaging as well as the fluoroscopic angles used intraoperatively for all 24 patients

View	Minimal	Maximal	Arc	Average
Preoperative CT inlet	7	37	30	20.5
Preoperative CT outlet	30	59	29	42.8
Fluoroscopic inlet	12	38	26	24.9
Fluoroscopic outlet	29	52	33	42.2
Postoperative CT inlet	8	31	23	19.4
Postoperative CT outlet	31	56	25	43.2

The minimal and maximal values are listed in addition to the corresponding angular arcs and averages

**Table 2:** Comparison of preoperative CT inlet and outlet measurements with the fluoroscopic angles used intraoperatively

View	Minimal difference	Maximal difference	Range of values	Average
Preoperative CT inlet compared to fluoroscopic inlet	−21	5	26	4.4
Preoperative CT outlet compared to fluoroscopic outlet	−9	7	16	0.45
Postoperative CT inlet compared to preoperative inlet	0	6	6	2.04
Postoperative CT outlet compared to preoperative CT inlet	0	7	7	2.54

**Table 3:** Summary of the measured inlet and outlet angles for the preoperative CT, intraoperative fluoroscopic views, and postoperative CT scan for each patient

Patient	Preop CT inlet	Fluoro inlet	Postop CT inlet	Preop CT outlet	Fluoro outlet	Postop CT outlet
1	13	22	15	47	40	40
2	25	30	23	41	50	45
3	19	20	22	47	45	47
4	37	32	31	44	42	44
5	23	20	23	39	40	42
6	9	30	8	44	43	44
7	18	26	19	59	52	56
8	19	21	20	45	40	44
9	7	12	8	46	40	42
10	22	30	25	38	42	40
11	10	19	8	46	41	47
12	27	31	25	42	42	39
13	32	28	28	47	50	52
14	21	21	18	30	29	33
15	17	20	16	45	40	40
16	24	30	23	43	51	44
17	12	18	9	43	42	41
18	21	27	20	41	45	46
19	10	15	8	56	51	55
20	22	30	21	42	39	44
21	21	27	21	41	40	43
22	28	29	25	30	35	34
23	32	38	31	42	40	44
24	24	23	19	30	38	31
Average	20.5	24.9	19.4	42.8	42.4	43.2
Range	7–37	12–38	8–31	30–59	29–52	31–56

The average and range of values is listed for each measurement

## Discussion

While some surgeons advocate relying on computer-assisted and navigated systems, it is common to utilize inlet, outlet, and lateral fluoroscopic images to safely instrument the posterior pelvic ring [1, 2, 7, 12, 21–23]. Obtaining quality intraoperative fluoroscopic images remains incredibly important and being able to correctly interpret the radiographic landmarks of the pelvis and their relationship to anatomical structures is mandatory [7, 24, 25]. In addition to the fracture pattern and displacement, preoperative CT scans demonstrate the osteology of each patient. Utilizing the preoperative CT, anticipated inlet and outlet angles can be measured and brought to the operating room to help the surgeon and radiology technician obtain accurate intraoperative imaging. In our series of 24 patients, the preoperatively measured inlet and outlet angles were within 5° and 1°, respectively, of the corresponding intraoperative fluoroscopic angles.

The anatomic variability of the pelvis has been well documented [14–16]. Figure 1 exemplify this variation and how the radiographic or fluoroscopic beam would need to be angled differently in each patient to obtain an ideal inlet view that parallels the anterior cortex of the S1 body. Traditionally, inlet and outlet radiographs were obtained by directing the beam 45° caudally and 45° cranially from the direct AP view [15, 26, 27]. This definition has evolved over time and several studies have since shown that the angles required to obtain inlet and outlet views differ greatly from this [15, 16]. Utilizing a similar measurement method as in our series, Graves et al. showed an ideal intraoperative inlet fluoroscopic view of 25° (21°–33°) and an ideal intraoperative outlet fluoroscopic view of 42° (30°–50°) [16]. Similarly in our series, the average ideal intraoperative inlet fluoroscopic view averaged 24.9° (12°–38°) and an average intraoperative outlet view to S1 of 42.4° (29°–52°). Standardized views do not account for the wide variability of the posterior pelvic ring. While erroneous placement of screws despite apparent appropriate screw positioning on intraoperative fluoroscopy has been documented, unintentionally utilizing incorrect imaging could lead to implant malpositioning and unintended iatrogenic injury to neurovascular structures [28]. While increasing the technical demands of iliosacral screw placement, attaining patient-specific non-orthogonal imaging leads to a more precise identification of the posterior pelvic ring anatomy [16]. The preoperative CT scan allows for the measurement of each patient’s individual posterior pelvic ring alignment. This preoperative measurement can be taken to the operating room and assist in obtaining accurate intraoperative fluoroscopic views. Correlating accurate intraoperative imaging with an accurate reduction, surgeon tactile feedback, and detailed knowledge of the available osseous fixation pathways will ultimately lead to safe implant positioning.

As recently demonstrated by Miller et al., the excessive fat density associated with morbid obesity makes visualization of the pelvic bony landmarks very difficult [13]. In fact, if the preoperative lateral CT scout view does not demonstrate identifiable landmarks, the intraoperative fluoroscopic lateral would also not be dependable. In such cases, the surgeon must have sufficient information and understanding from the preoperative CT imaging and the intraoperative fluoroscopic inlet and outlet views to proceed safely without a confirmatory lateral view. Obtaining adequate fluoroscopic views is challenging in the obese patient population and having a detailed preoperative plan with the knowledge of the anticipated intraoperative views is invaluable.

Obtaining suboptimal views by malrotation of the C-arm has been shown to effect the safe placement of iliosacral screws. Wolinsky et al. demonstrated that by rotating the C-arm >8° towards the foot away from the ideal inlet view, an out-the-back wire can appear to be contained within the bony sacrum [29]. Unknowingly relying on imperfect views could lead to the placement of unsafe iliosacral screws leading to serious neurological or vascular injury. By performing preoperative measurements that closely correlate with the expected intraoperative angles, a surgeon can minimize the incidence of obtaining imperfect images during the procedure. The inlet view appears to have more variability between the preoperative and intraoperative measurements. Typically, an ideal fluoroscopic inlet will have a thickened cortical density that corresponds to the overlap of the S1 anterior cortex of the S1 body. At times, the S1 and S2 body will have the same orientation and a very thick density can be appreciated. Often though, the S1 and S2 body will have a different orientation in the sagittal plane and such a distinct cortical density is not appreciated. When this variability is present, multiple views can make the inlet view appear to have an appropriate density when it is really just erroneous interpretation that does not truly correspond to the correct anterior cortical overlap. Although the preoperative measurements are not flawless, it is one more tool a surgeon can use towards performing a safe and successful procedure.

The limitations of this study include the small sample size. Our goal was not to compare patients in separate cohorts, but to see if a measuring technique was reproducible to a single surgeon as well as applicable intraoperatively. One surgeon made each angle measurement both preoperatively and postoperatively and these measurements only differed by approximately 2° and statistically showed a high degree of intraobserver correlation. At our institutions, these measurements have been anecdotally very reproducible between surgeons. It would also be beneficial to validate good interobserver reliability to these specific measurements. Although very close, this overall method is not without potential for error. One potential source for error stems from patient positioning. If the lumbosacral bump is placed either too proximally or distally, the patient will have increased or decreased lumbar kyphosis, which will directly affect the translation of the preoperative measurement into the operating room. One patient in this series had a 20° range of variation from the preoperative and intraoperative inlet. It is hypothesized that malpositioning could have contributed to this as other patients did not have such a high degree of change. Another source of error could stem from misreading of the measurement intraoperatively by the fluoroscopic technician. The measurements as dictated in the operative notes were reported to the surgeon by the radiology technician and not directly visualized by the surgeon. Different fluoroscopic machines display the degree of inlet and outlet cant with varying degrees of detail. For instance, some fluoroscopic machines only have a marking every 15°. This could easily be misinterpreted, documented incorrectly, and ultimately lead to an improper reading. Care should be taken to identify the correct measurement and correlate this with the necessary identifiable osseous landmarks to obtain the correct view.

All patients had iliosacral screws placed in a supine position in this study. In theory, the same method could be used to preoperatively plan with the patient in a prone position. The surgeon would have to consider how the prone positioning would affect the lumbosacral alignment. In comparison to supine positioning of the preoperative CT scan, the bolsters placed for appropriate positioning and padding may alter the orientation of the pelvis in space. This potential change could be assessed on a lateral fluoroscopic view if possible and the difference accounted for. This was not investigated in this study as no patient was placed prone during this period.

In conclusion, there is a significant amount of anatomic variation of the pelvis, particularly the posterior pelvic ring. CT imaging is invaluable in demonstrating the injury patterns, detecting differences in sacral morphology, and displaying the available osseous fixation pathways. Although not flawless, preoperative CT sagittal reconstruction images allow for appropriate preoperative planning for anticipated intraoperative fluoroscopic inlet and outlet views within 5°. Obtaining quality intraoperative images can be difficult in certain patient populations and clinical situations. Possessing an in-depth understanding of each patient’s pelvic anatomy and correctly interpreting the corresponding bony landmarks intraoperatively is paramount. Having knowledge of the desired intraoperative views preoperatively can prepare a surgeon, aid in efficiently obtaining the correct views intraoperatively, and ultimately assist in the placement of safe iliosacral screws.
